# What Makes a Mother? Investigating Maternal Success in Ex Situ Cheetahs

**DOI:** 10.1002/zoo.21894

**Published:** 2025-04-13

**Authors:** Sian Barr, Yu‐Mei Chang, Lars Versteege, María Díez‐León

**Affiliations:** ^1^ Royal Veterinary College, University of London London UK; ^2^ Zoological Society of London London UK; ^3^ Safaripark Beekse Bergen Hilvarenbeek The Netherlands

**Keywords:** *Acinonyx jubatus*, behaviour, offspring survivorship, zoological collections

## Abstract

Understanding the factors influencing the likelihood of breeding success is essential to the sustainable management of ex situ populations. Using keeper questionnaires and studbook data, we investigate maternal success in Southern Cheetahs (*Acinonyx jubatus jubatus*) in relation to life history and husbandry factors. Maternal success was measured using five maternal success indicators: (i) overall litter size; (ii) proportion of liveborn cubs in a litter; (iii) proportion of liveborn cubs raised by their mother surviving the 6‐week neonatal stage; (iv) proportion of liveborn cubs raised by their mother surviving to 12 months; and (v) absence of maternal neglect. Cheetahs rep the feeding of ruminant meat and carcasses to cheetahs was found to be significantly related to a larger average litter size while the practice of starve days was associated with a higher stillbirth rate. Females who were moved to new enclosures for the pregnancy, lactation and parenting period also had poorer maternal success. While enrichment provision did not appear to result in a more positive mothering outcome, feederballs and catnip use were associated with lower mothering success, highlighting the importance of evidence‐based practice in ex situ collections.

## Graphical abstract

Feeding and diet may play a part in maternal success. Cheetahs who are reported to feed on ruminant meat have larger litters of cubs than female cheetahs who are not, while female cheetahs who are reported to experience starve days have a higher incidence of stillbirths.

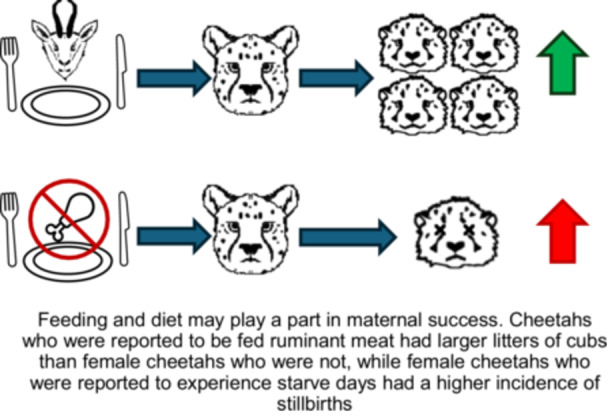

## 1 Introduction

To maintain closed ex situ animal populations, breeding success is a fundamental requirement. Without this, these populations are not viable long term, yet the nature of ex situ animal interactions presents challenges for breeding plans (Lacy 2013). Optimising successful breeding practices is therefore vital to ex situ collections.

Introducing animals for breeding with low mean‐kinship rankings is not an automatic recipe for success: animal mating and parenting is governed by a myriad of factors, including behaviour, environment and individual preference (Asa et al. 2011; Lees and Wilcken 2009; Martin‐Wintle et al. 2019, 2015; Willoughby et al. 2017). While genetic representation remains important and can be managed through breeding schemes such as the European Endangered Species Programmes (EEPs), the sustainability of these schemes hinges on animals matched as a priori suitable pairings by the EEP to actually conceive and raise offspring to maturity (Asa et al. 2011; Conway 2010; Forti 2019; Lees and Wilcken 2009). Currently, the success of such pairings remains low across taxa, with up to 80% of pairs failing to breed in some species (reviewed in Martin‐Wintle et al. 2019). Understanding the factors that influence likelihood of breeding success is essential in engineering circumstances that encourage this success.

In mammals, it is well recognised that maternal health and behaviour impacts on the short‐ and long‐term life of their offspring. Attentive maternal behaviour in rats (*Ratus norvegicus*) toward offspring is linked to the increased memory and learning capacity of those offspring, while poorer maternal care increases the risk of offspring developing abnormal repetitive behaviours (ARBs) (Latham and Mason 2008; Liu et al. 2000). Maternal experience also impacts offspring survival: younger inexperienced Geoffroy's cat (*Leopardus geoffroyi*) mothers have more difficulties in raising liveborn litters to independence than older, experienced females and in Polar bears (*Ursus maritimus*), younger females are more likely to lose cubs in their first few months of life (Folio et al. 2019; Foreman 1997). It is critical for the sustainability of viable ex situ populations for an animal's offspring to be raised to independence and to go on to reproduce. The significance of the successful reproduction is amplified in species of notable genetic homogeneity, such as the cheetah (*Acinonyx jubatus*), as successful breeding by desired individuals is required to preserve existing genetic diversity within the population (Charruau et al. 2011; Forti 2019; Lees and Wilcken 2009; Marker and O'Brien 1989; Willoughby et al. 2017). Without this, the existing ex situ cheetah population will be less adaptable and may not be viable in future decades (Lees and Wilcken 2009). Cheetahs are furthermore a useful study species due to well documented public domain studbooks in addition to the existence of two subspecies managed by EEPs.

For cheetahs, maternal success requires productive mating, pregnancy, parturition and parenting cubs to independence (Alves et al. 2018). While both sexes play a role in cub conception, adult females typically raise their cubs alone, nurturing their offspring for approximately 18 months in the wild (Durant 2000; Kelly et al. 1998; Laurenson 1994). Ex situ, cubs are not reliant on their mothers’ hunting skills and vigilance against predators as their wild counterparts are, yet the role of mothers in ex situ animals remains relevant to cub survival (Caro 1987; Laurenson 1994, 1993). For example, maternal ‘neglect’ was attributed as a contributing factor to cub mortalities in European cheetah collections in the 1990s, though the meaning of this term was not defined by the authors (Marker and O'Brien 1989; Sengenberger et al. 2018). Negligence itself is a subjective term despite appearing frequently in cheetah breeding literature. The failure of the mother cheetah to perform the necessary levels of feeding, grooming, cleaning and interaction with cubs could be considered neglect in mother cheetahs, as these behaviours allow cubs to thrive. In one paper, neglect was considered a distinct behaviour separate from cub abandonment or more active aggressive behaviours such as cannibalism, though the three categories often overlap (Laurenson 1993). Neglect, cub abandonment and aggression towards cubs are behaviours sometimes displayed by ex situ cheetahs but is rarely documented in the wild unless prey is scarce (Laurenson 1993). Other evidence also supports the importance of cheetahs’ maternal behaviour on the short‐ and long‐term lives of their offspring, including their reproductive success. Male cheetahs’ hand‐reared by humans (*Homo sapiens*) mature into animals who produce fewer offspring than their mother‐raised counterparts which is theorised to be due to failure by these individuals to interpret mating cues (Hampson and Schwitzer 2016). This finding is likewise noted in hand‐reared female domestic cats (*Felis catus*) (Mellen 1992). While it can be hypothesised that increased maternal negligence is likely to be negatively associated with maternal success in ex situ animals, investigation is required to confirm if this is true in cheetahs.

In terms of life history, female reproductive success has been shown to have a genetic component, with litter size appearing to be a heritable characteristic, suggesting matriline could influence ex situ maternal success (Bertschinger et al. 2008; Kelly 2001). Litter size is also impacted by age; uterine health declines with age, resulting in a less hospitable embryonic environment and older cheetahs have previously been shown to produce smaller litters (Augustus et al. 2006; Crosier et al. 2011) Captive females are reported to produce most litters between 4 and 9 years old and while age is not a proven factor of offspring survivorship in cheetahs (Sengenberger et al. 2018), in wild leopards (*Panthera pardus*) litter size and cub survival rate decline in older females (Balme et al. 2013) and in tigers (*Panthera tigris*), reproductive parameters decline as females age (Tidière et al. 2021). Survivorship of litters requires further investigation. Age does not appear to influence adult personality (Sengenberger et al. 2018; Wielebnowski 1999), which research has also shown to affect cheetah breeding success: cheetahs scoring higher for tense‐fearful personality traits are less likely to be successful breeders, though this is difficult to confirm without exposing all females in an experiment to the same male (Wielebnowski 1999). Cubs born to primiparous litters and quintiparous litters have been indicated to gain less weight than tertiparous counterparts and, since good weigh gain in cheetah cubs positively predicts survivorship, this suggests maternal parity impacts the likelihood of young cheetah cubs to thrive (Beekman et al. 1999). It is less clear whether maternal parity influences the success of raising older cubs to maturity and, as very young females are unlikely to be carrying their fifth litter, it is difficult to differentiate parity effects from maternal age affects.

Further, maternal success varies significantly between institutions in this species, suggesting that variations in husbandry factors may impact successful cheetah breeding (Marker et al. 2018; Sengenberger et al. 2018; Wielebnowski 1996). This may come down to husbandry factors such as training or environmental enrichment. As appropriate enrichment may vary according to an individual animals natural biology, life stage and needs, useful environmental enrichment can be considered an “improvement in the biological functioning [such as reproductive success] of captive animals resulting from modifications to their environment” (Newberry 1995). Positive environmental enrichment has been shown to alter captive cheetah behaviour, for example, and is considered to be a positive for the animals’ wellbeing, although evidence showing an influence on cheetah maternal behaviour is limited (Quirke and O'Riordan 2015, 2011; Woc Colburn et al. 2018). In other mammals, effects of enrichment on maternal behaviour are documented for species with altricial young. Enrichment in American Mink (*Neovison vison*) benefits productivity, improves nest building and reduces stereotypic behaviour that might detract from maternal behaviour while enrichment provision in rats increases complex mothering behaviour (Díez‐León and Mason 2016; Meagher et al. 2014, Cutuli et al. 2015). Interaction between humans and animals appears to be beneficial to the breeding success of small, ex situ felid species and trained fishing cats (*Prionailurus viverrinus*) have been shown to have greater copulatory success than untrained counterparts (Fazio et al. 2019; Mellen 1991; Mellen and Shepherdson 1997). This is relevant also for cheetah because behavioural training is used in some institutions as a method of animal management, particularly in reducing the invasiveness required for medical procedures (Woc Colburn et al. 2018), yet not necessarily consistently across facilities. Some authors have suggested trained animals may be less stressed than untrained animals (Woc Colburn et al. 2018). Whether these factors affect the breeding success or maternal behaviour of cheetahs remains unknown. As physiological stress has been negatively linked to reproductive success and cheetah health, it could be hypothesised that untrained cheetahs with no environmental enrichment may have less maternal success due to increased stress levels (Woc Colburn et al. 2018).

The enclosure itself presents another environmental factor that varies between institutions, with designs and resources provided motivated by tradition or popularity with visitors in addition to animal impact (Ahlrot 2016; Clubb and Mason 2007). Within‐enclosure platforms, for example, appeal to the public by allowing animals be seen but also appear to appeal to felid species as a preferable location (Ahlrot 2016). Cheetahs are known to defecate on such platforms, though the significance of this behaviour or whether platform use has any impact on welfare or breeding success remains unexplored (Ahlrot 2016; Lyons et al. 1997). The EEP recommends allowing breeding cheetahs to overlook prey species in neighbouring enclosures, though there is limited empirical evidence to define how successful this tactic is (Sengenberger et al. 2018; Woc Colburn et al. 2018). The EEP also recommends that cheetah mothers are offered nesting boxes in order for increased privacy to reduce stress (Sengenberger et al. 2018; Woc Colburn et al. 2018). If mothers offered concealment are more successful, it may be that those with exposed enclosure features such as platforms also have their success impacted but this requires exploration. The EEP also recommends that breeding female cheetahs should be off‐exhibit and not co‐habiting with other cheetahs, and therefore collection management is faced with a dilemma between best practice and the benefits of display, with some institutes opting to move gravid mothers or new young off‐show during breeding times (Sengenberger et al. 2018; Woc Colburn et al. 2018). The effect of this is understudied in cheetahs but in Geoffroy's cats, a historically successful mother who was moved to a new enclosure for parturition and parenting was noted to show aberrant behaviour towards her kittens (Foreman 1997). This behaviour vanished once the mother and her litter were returned to her familiar enclosure (Foreman 1997). In American mink, movement of pregnant females did not significantly affect faecal cortisol nor have a significant correlation with litter size or growth (Schou et al. 2019). Exploring the relationship between enclosure elements and successful breeding would assist collections in making practical choices in housing their breeding cheetahs.

Differences in nutrition across institutions might also be at play. Free‐roaming cheetahs hunt and consume vertebrate prey, consuming the whole carcass, but whole‐carcass feeding is not universal practice within zoological collections (Sengenberger et al. 2018). This is notable because wild cheetah reproduction peaks in correlation with ungulate calf surplus, suggesting feeding cycles may play a role in cheetah breeding (Laurenson et al. 1992). Dietary deficiencies and imbalances have been shown to impact both hepatic health and ovarian activity in cheetahs (Davidson et al. 1986; Setchell et al. 1987). Similarly, a fatty diet during pregnancy and lactation has been found to be associated with a reduction in maternal care in rats (Connor et al. 2012). Understanding if diet impacts maternal success in cheetahs warrants research but is not straightforward. In European zoos, beef, horse, poultry, lamb, goat and rabbit are commonly fed to cheetahs (Whitehouse‐Tedd et al. 2015). Cheetah diet food items vary amongst institutions due to item accessibility and managerial preferences and while whole‐carcass feeding is recommended, it is not universally practiced and would benefit from additional research to support its use (Sengenberger et al. 2018; Whitehouse‐Tedd et al. 2015). Feeding patterns, too, vary by institution. Starve days, designed to mimic wild fast‐gorge feeding patterns, have been adopted by some cheetah holders due to the bodyweight and digestive benefits identified in captive lions fed this way (Altman et al. 2005; De Cuyper et al. 2019; Sengenberger et al. 2018), yet their impact on breeding success remains unknown and this practice is not universal.

Lastly, within EEPs, breeding recommendations are based on genetics as opposed to animal preferences, yet female cheetahs are known to display mate choice preferences in regards to copulatory partners (Sengenberger et al. 2018; Ziegler‐Meeks 2009). Mate choice is effective in improving offspring survival in giant pandas (*Ailuropoda melanoleuca*) and lack of this has been suggested as a reason for poor breeding success in cheetahs (Asa et al. 2011; Augustus et al. 2006; Martin‐Wintle et al. 2015). A cheetah's response to a prospective male can be predicted by her reaction to exposure to his urine but whether mate choice translates to maternal success beyond copulation is unknown (Mossotti 2010; Mossotti et al. 2018).

With so many potential influencing factors, the aim of this paper is to identify what, if any, areas warrant more specific investigation to identify the best conditions and circumstances required for cheetah maternal success, while providing initial testing of some of the hypotheses outlined above and generating new ones. In conservation research, where funds are likely to be limited, this information could be of use in selecting sensible avenues of exploration.

## 2 Materials and Methods

The European Association for Zoos and Aquaria (EAZA) manages Northern and Southern cheetah subspecies (*A. j. soemmerringii* and *A. j. jubatus*, respectively) as separate populations (Marker and O'Brien 1989; Sengenberger et al. 2018). This study focuses on the Southern subspecies as it is this population that had the greater accessible breeding data. The target population was female Southern EEP cheetahs over 18 months of age who had produced at least one litter between 2009 and 2019, including those with stillborn litters. A survey was sent to 84 zoos holding this subspecies by the EEP coordinator (LV). Sixteen zoos holding eligible female cheetahs participated. A further 19 zoos responded to confirm they were ineligible as they had not produced litters in the timeframe. The remaining 45 zoos did not reply. A 42% response uptake with a 19% data generation is lower than comparable zoo studies but consistent with general organisation‐aimed questionnaire studies (Baruch and Holtom 2008; Little et al. 2016; Tanaka and Ogura 2018).

Maternal success was defined as conception, pregnancy to term, parturition and parenting of offspring to independence and/or was indicated by survivorship of offspring. This was measured using five maternal success indicators (MSI): (i) total number of cubs born in a litter; (ii) proportion of liveborn cubs in a litter; (iii) proportion of liveborn cubs raised by their mother surviving the 6‐week neonatal stage (abbreviated to short term mothering success or STMS), by which time cubs are mobile and starting solid food; (iv) proportion of liveborn cubs raised by their mother surviving to 12 months (abbreviated to long term mothering success or LTMS), which is the earliest age of independence in wild cubs; and (v) absence of maternal neglect (Kelly et al. 1998; Sunquist and Sunquist 2017). To minimise the subjectivity of the term “neglect”, neglect was defined in the questionnaire as the failure of the mother cheetah to perform the necessary levels of feeding, grooming, cleaning and interaction with cubs that resulted in temporary or permanent human intervention.

An online questionnaire was created using SurveyMonkey (SurveyMonkey 2019) and emailed to member zoos (see Supporting Information S1). The questionnaire was designed to be completed by zookeepers and animal managers who were directly involved with the cheetahs within their organisation. Before launch, a pilot questionnaire was sent to the Southern Cheetah EEP coordinator (LV) and the Zoological Society of London Whipsnade Zoo Africa Region Team Leader for their input. This was to ensure that the questionnaire was user‐friendly, applicable and relevant to modern cheetah keeping. Edits were made following the feedback of these stakeholders to specify husbandry practices. For example, following discussion with keepers, it was found that some cheetah enclosures include natural terrain elevation and so this was included alongside provided platforms. Similarly, different enrichment types were included following keeper discussions because it became apparent that enrichment could be interpreted differently.

The questionnaire combined both multiple choice questions and open‐ended questions. Questions were asked regarding the history and husbandry of female cheetahs. Questions were also asked surrounding MSIs. To avoid definition drift between participants, terms were defined within the relevant sections of the questionnaire (Fox et al. 2003). Neglect was defined as aforementioned. Aggression was defined as biting, slapping, injuring or killing behaviour aimed towards cubs.

### 2.1 History Factor Questions and Data

To assess potentially influential history factors, questions were asked regarding maternal age and parities. The International Studbook (ISB) number of each female was cross‐referenced with studbook reproduction reports over the relevant years to identify if any female had bred at previous organisations, ensure the reliability of parity data, and trace the matriline of the cheetahs (Marker 2005–2008, 2010–2013). The questionnaire asked if females were primiparous and for maternal age at litter birth.

### 2.2 Husbandry Questions

Included in the questionnaire were questions regarding diet, enclosure, enrichment and mate choice. Following discussions with cheetah keepers, who indicated that meat from birds and hoof stock are typically used as part of an ex situ cheetah diet, we asked if avian or ruminant meat was part of the cheetah's usual diet and whether starve days were included in the husbandry routine. A food item was considered part of a cheetah's regular diet if offered at minimum once fortnightly. Starve days were defined as a day cycle where cheetahs were provided with no food items at all, including treats or food‐based enrichments.

Regarding usual enclosure, the enclosure the cheetah lived in when non‐breeding, participants were asked about the visibility of other cheetahs to the female cheetah from her usual enclosure, the presence or absence of enclosure elevation or platforms, and whether the mother was moved from her usual enclosure for pregnancy and parenting. ‘Usual enclosure’ was considered the space(s) in which a cheetah lived when not pregnant or parenting; this was stated specifically because zoos may move animals to better display cubs, an action which has been associated with poorer maternal care in other species (Foreman 1997). Elevation was divided into keeper‐manufactured artificial features between 0.5 and 2.0 m tall and elevated natural terrain to ensure that enclosures incorproating pre‐existing hills were also captured by the questionnaire. Regarding breeding, questions were asked about the level of mate choice offered to the female and whether husbandry alterations were undertaken when a female was pregnant, lactating or raising cubs. This was included to ensure that any alterations to husbandry around breeding in comparison to non‐breeding times were identified. Animals who were introduced to their mate after showing interest at the sight or scent of him or through accidental introduction were considered to have been offered a mate choice.

Questions also included whether the cheetahs had human interaction through free‐contact or protected contact and if they were trained, including if they were trained for veterinary procedures as the latter could impact on ability to perform health checks routinely. Protected contact was defined as physical interaction between keepers and cheetahs through a barrier, while free‐contact was defined as keepers entering enclosures with free‐roaming cheetahs for husbandry or to interact. This was included as it was initially unknown if this was a common husbandry practice. Human presence during labour in giraffes (*Giraffa camelopardis*) is linked to increased maternal rejection but contrastingly, in wolverines (*Gulo gulo*), allowing animals to free roam in an enclosure alongside keepers performing maintenance was associated with more successful breeding (Loberg et al. 2020; Siciliano‐Martina 2020).

### 2.3 Enrichment

Enrichment was defined in the questionnaire as management provisions applied to a cheetahs’ schedule in order to provide a positive benefit to the animal, which might be physical, behavioural or psychological (Shepherdson et al. 2012). Following discussion in the pilot stage, it was noted that a range of enrichments may be offered to this species (Mark Holden, pers. comm.). Consequently, we investigated each type of enrichment individually as the benefits conferred to the cheetah may not have been consistent across all varieties of enrichment. This incorporated questions about specific types of enrichment including feederballs, lures, novel scents, novel objects, catnip, the presence, and frequency of enrichment in husbandry practices, and enclosure rotation.

The questionnaire was launched on June 14th 2019, with follow‐up reminder emails sent on June 24th 2019 and July 2nd 2019. The questionnaire circulated for 46 days and was closed on July 30th 2019.

### 2.4 Statistical Analyses

Raw data was exported from SurveyMonkey, processed into quantitative form in Microsoft Excel (Microsoft 2019), cross‐referenced with International Studbook data and inputted into SPSS (SPSS Statistics v.22.0, IBM 2016) for analysis. Type I error rate was set at 5%.

Generalised estimating equations (GEE) were used to investigate the effects of life history and husbandry variables on MSI variables (Pekár and Brabec 2018). Outcome variables were litter size, proportion of stillbirths per litter, STMS, LTMS, and if negligent behaviour was reported. The GEE approach was selected as it has the flexibility to incorporate both cheetahs with single and repeated observations (i.e., repeated measures across years due to multiple litters) using exchangeable working correlation structure. Predictors tested were whether the female was primiparous, female cheetah's age, generations from the wild via the matriline, proximity to other cheetahs, enclosure elevation, if cheetahs moved enclosure to breed, diet including ruminant meat, diet including avian meat, starve days, training, mate choice, if female was enriched and types of enrichment. Additionally, in the GEE where litter size was the outcome variable, a Poisson distribution was used, and results reported as rate ratio and 95% confidence interval (CI); for the rest of the GEE models, binomial distributions were used, with results reporting odds ratios and 95% CIs.

Analysis followed a two‐step approach. Firstly, univariable GEEs were run to evaluate each success indicator against each potentially influential factor. Factors identified as significant in the univariable GEE were subsequently run in a multivariable GEE model and backward elimination model selection was used to identify independent predictors for each success indicator.

## 3 Results

Two litters were shown aggression by their mothers. Aggression was initially believed to be a possible indicator of lack of maternal success but was excluded from analysis due to small sample size. Two cheetahs reportedly cannibalised their whole litter before keepers could confirm how many cubs were born and if any were initially alive. One cheetah produced live conjoined twins (the first case reported for cheetahs to our knowledge); they died before reaching 1 week old but for relevant analyses (litter size and number of liveborn cubs), they were categorised as one cub. Three litters (14 cubs) still with their mothers were younger than a year old at the time of survey completion which meant that LTMS could not be calculated for those litters. Institutional variations in diets meant organs, bones, whole carcasses and lagomorph carcasses as food items could not be tested individually due to limited representation.

Forty litters born to 22 dams between 2009 and 2019 in eight countries were included in analyses. Litter size ranged from one to six cubs, with an average litter size of 3.7 cubs and an average liveborn litter size of 3.0 cubs. A total of 142 cubs were born in participating facilities, of which 117 were liveborn (82%). Of cubs born, 86 cubs (60.5%) were still alive and were being parented by their own mother by 6 weeks of age and 62 cubs (43.6%) were still alive and being parented by their mother up to and beyond 1 year old. Eleven litters were identified as having been shown minor or major maternal insufficiency, which we have referred to as negligence by their mother, that resulted in temporary or permanent human intervention or cub demise. Five females (22.7%) did not raise any cubs to 6 weeks of age, of which one did not produce any liveborn cubs. Seven females (31.8%) produced at least one stillborn cub. Fourteen females (63.6%) mothered at least one liveborn cub beyond 1 year of age.

The dataset size available for each univariable GEE analysis varied, as some questions were irrelevant for some cases, such as the long‐term mothering questions for cheetahs whose cubs had all died at birth. Not every participant chose to answer every question, causing further sample size fluctuation (Supporting Information S1: Tables Sa–e).

### 3.1 Individual History Effects on Mothering

When tracing the matriline, females were found to be between five and eight generations removed from their wild female ancestors. Of the three females for whom we could not confirm their maternal line origin, one cheetah was the fifth generation descendent of a female of unknown origin and two females were themselves of unknown origin. While generations from the wild had no influence on litter size, negligence, STMS or LTMS, there appeared to be a tendency for females further removed from the wild to have a lower proportion of stillbirths (OR: 0.240, CI: 0.055–1.053, *p* = 0.059, Supporting Information S1: Table Sb).

The average maternal age at a litter's birth was 5.3 years (range: 2–9 years). Seventeen litters were confirmed first‐born litters. The average age for a first litter was 4.1 years old. The youngest primiparous cheetah was 2 years old and the eldest 7 years old. Average number of litters per cheetah was 1.8 litters, with 10 females (45.4%) producing just one litter and one female (4.5%) producing four litters. Age was negatively associated with litter size in univariable analysis (RR: 0.935, CI: 0.900–0.971, *p* < 0.001) but this disappeared when modelled with husbandry practices. It is relevant to note that age was correlated with other variables, including feeding of ruminant meat and not using feederballs or novel scents as enrichment, due to small size. Being primiparous was not found to effect MSI (Supporting Information S1: Tables Sa–e).

### 3.2 Husbandry Effects on Mothering

Husbandry routines described by keepers were found to remain consistent for individual females across all litters but varied amongst individual cheetahs and between zoos. All female cheetah lived within 5 km of at least one male cheetah. Eight females lived within sight of one or more males and eight females could always see other females. Six cheetahs had non‐offspring companions all or part of the time but eight lived alone when not parenting, two of which were out of sight of other cheetahs. Constant proximity to other cheetahs of either sex outside of breeding season had no significant effect on MSI (Supporting Information S1: Tables Sa–e). There was a weak tendency for females housed out of sight of males raising more liveborn cubs to 6 weeks (Supporting Information S1: Table Sa).

Every cheetah had some form of elevation in their enclosure, of which 10 had natural terrain elevation (e.g., hills) and 12 had artificial platforms, so it was not possible to measure if elevation itself influenced maternal success. Provision of artificial elevation in usual enclosure rather than natural elevation showed no effect on STMS, LTMS, or neglect but data was insufficient to test for effect on stillbirths (Supporting Information S1: Tables Sa–e). Cheetahs housed with artificial elevation platforms produced smaller litters, an effect that persisted after inclusion in multivariable analysis (Table 1).

**Table 1:** History and husbandry factors influencing litter size in cheetahs.

Variables	Multivariable analysis		
	Rate ratio (95% confidence interval)	*n*	*p*‐value
Mother cheetah receives ruminant meat every fortnight at minimum	1.678 (1.594–1.767)	29	<0.001
Novel scents are either never/rarely used as enrichment^a^	0.698 (0.674–0.723)	29	<0.001
Feederballs are not used as enrichment	1.674 (1.355–2.067)	29	<0.001
Artificial elevation platforms provided in enclosure	0.849 (0.786–0.917)	29	<0.001
Novel objects are either never/rarely used as enrichment^a^	0.469 (0.357–0.616)	29	<0.001

a Where “rarely” refers to something practiced less frequently than monthly.

**Figure 1:**
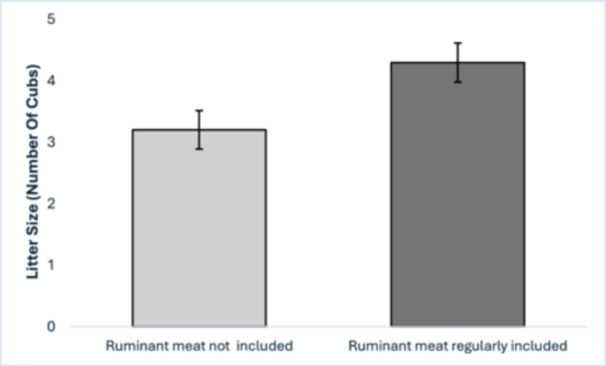
Effect of provision of ruminant meat and carcasses in a female cheetah's regular diet on litter size. Bars represent mean litter sizes; error bars represent standard error.

The diets of the cheetahs surveyed included organ meats, whole carcasses, bones, avian meat and ruminant meat. Avian meat was included in the diet of 13 cheetahs but had no influence on MSI. Ruminant meat was included in the diet of 11 cheetahs. Ruminant meat inclusion in a cheetah's diet had no effect on stillbirth rate, neglect, STMS, or LTMS but it did affect litter size, with average litters being one cub larger in females who received ruminant meat in their regular diet (Figure 1, Table 1).

**Figure 2:**
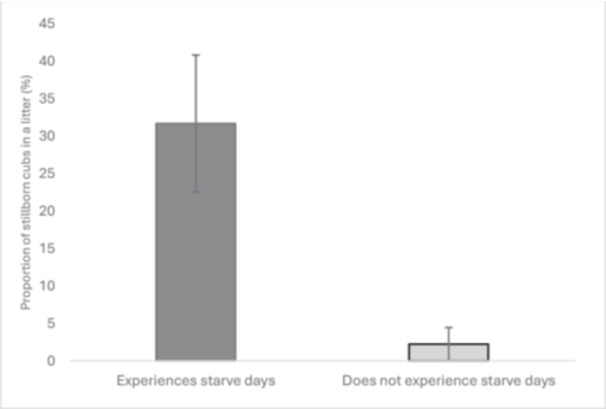
Effect of implementation of starve days in the routine feeding practice of female cheetahs on the percentage occurrence of stillbirths per litter. Bars represent mean; error bars indicate mean plus/minus standard error.

Frequency of starve days per week ranged from zero to every other day, with one or two starve days a week the most common starve protocol. Thirteen cheetahs received one or more starve days weekly during their regular feed schedule. The inclusion of starve days was associated with a greater proportion of stillborn cubs (Figure 2, Table 2) but had no effect on total number of cubs born, neglect, STMS, or LTMS. Females who experienced starve days saw 29.4% of cubs born as stillbirths, whereas 1.6% cubs were stillborn to mothers who were fed every day.

**Table 2:** History and husbandry factors influencing the occurrence of stillbirths in cheetahs.

Variables	Multivariable analysis		
	Odds ratio (95% confidence interval)	*n*	*p‐*value
Mother remains in her usual enclosure during pregnancy and parenting	0.094 (0.021–0.428)	37	0.002
Starve days practiced^a^	14.640 (1.117–191.810)	37	0.041

a Starve days practiced during routine husbandry. Data insufficient to test starve days practiced during pregnancy and parenting specifically.

Two cheetahs were managed in free‐contact, but both zoos reported that keepers did not actively seek out physical contact with cheetahs when entering the enclosure. Thirteen cheetahs were managed through protected contact. Seven cheetahs were managed with zero physical contact with humans, though verbal interaction was permitted. Thirteen cheetahs received some degree of training as part of their general husbandry, of which eight were trained to present for and tolerate more intensive veterinary procedures. Crate training, crush cage and recall were most common. In comparison to untrained cheetahs, trained cheetahs had no significant difference in litter size, STMS, LTMS, or negligence (Supporting Information S1: Tables Sa–e). This remained the case when comparing cheetahs trained for more intensive veterinary procedures.

**Figure 3:**
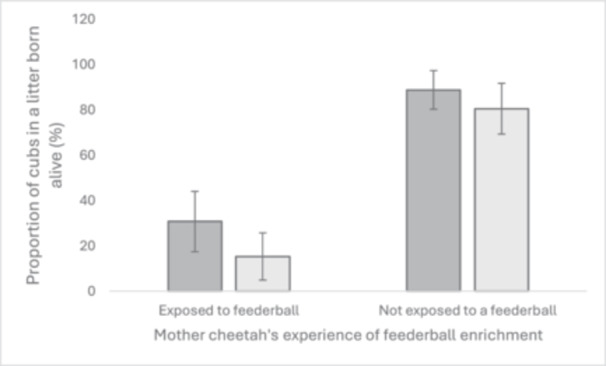
Mean percentages of liveborn cubs raised to 6 weeks (short‐term maternal success, STMS, indicated in dark grey) and to 1 year and beyond (long‐term maternal success, LTM, indicated in light grey) in female cheetahs who receive feederball enrichment compared to cheetahs who do not. Bars represent mean; error bars indicate standard error.

Twenty cheetahs received enrichment when not pregnant or parenting, though for some animals this was less than monthly. Seven cheetahs received enrichment daily. Enrichments used included feederballs (6 zoos), mechanical lures (3 zoos), fishing rod lures (2 zoos), novel scents (10 zoos), novel objects (11 zoos), catnip (*Nepeta cataria*) (6 zoos) and enclosure rotation with other animals, including different species. Laser‐pointers and audio enrichments were not used for enrichment for any animals in this study. Cheetahs who were familiar with enrichment when not pregnant or parenting showed no maternal success differences to those unaccustomed to enrichment MSI. When enrichment types were tested individually, however, maternal exposure to a feederball during usual husbandry was found to significantly affect STMS and LTMS; feederball‐enriched cheetahs raised less cubs to independence (Figure 3) and produced smaller litters (Table 1) than cheetahs who were never exposed to feederballs. Novel scent and novel object use were both positively associated with litter size, with cheetahs enriched with novel scents and/or novel objects producing bigger litters than those who were not (Table 1).

While the use of catnip as an enrichment had no effect on litter size, stillbirths, STMS, or LTMS, animals exposed to catnip were more likely to be reported by keepers to be negligent mothers compared to those who were not (Table 3). Negligence was reported in 46.1% of litters whose mother had experienced catnip enrichment in comparison to 21.4% of litters whose mother had never been enriched with catnip. The data did not capture how close the use of this enrichment was to the female's pregnancy, parturition and parenting.

**Table 3:** History and husbandry factors influencing the occurrence of keeper‐reported negligence in cheetahs.

Variables	Multivariable analysis		
	Odds ratio (95% confidence interval)	*n*	*p‐*value
Mother remains in her usual enclosure during pregnancy and parenting	0.144 (0.040–0.525)	27	0.003
Catnip is occasionally used as an enrichment device	6.381 (4.042–10.075)	27	≤ 0.001

*Note:* *Starve days practiced during routine husbandry. Data insufficient to test starve days practiced during pregnancy and parenting specifically.

Level of mate choice provided (excluding unplanned mating) varied between organisations. Of 22 females, 16 (28 litters) were offered no mate choice, while eight (12 litters) were given choice. Mate choice or lack thereof was found to have no impact on litter size, negligence, STMS, or LTMS (Supporting Information S1: Tables Sa,c–e). Data was insufficient to test for effects on stillbirths.

Nine cheetahs (18 litters) were transferred to a new enclosure specifically for parenting, though the data did not capture at which stage of pregnancy the enclosure transfers occurred. Thirteen females (22 litters) remained within their usual enclosure throughout the breeding process. Moving to a new enclosure had no effect on total number of cubs, STMS or LTMS (Table 1 and Supporting Information S1: Tables c,d) but cheetahs who moved were more likely to be reported by keepers as showing negligent mothering behaviour than those who were not (Table 3) and had a higher rate of stillbirths (Table 2). Of litters whose mother was pregnant and parented in her usual enclosure, 10.5% experienced maternal negligence as perceived by their keepers. In contrast, 50.0% of litters were perceived to be shown negligence when their mother had been transferred to a specific new enclosure for pregnancy and parenting.

The approach to providing a female cheetah with enrichment during parenting varied between organisations. Some zoos increased enrichment provision to females during the period when they had cubs. Others stopped enrichment during this time, stating that they considered cubs to be enrichment enough for females. When pregnant and parenting, thirteen cheetahs (25 litters) received enrichment. This was found to have no significant effect on litter size, stillbirths, STMS or negligence (Supporting Information S1: Tables Sa,b,c,e).

## 4 Discussion

This study aimed to identify factors that could be predictive of successful mothering in cheetahs by surveying current husbandry practices in organisations breeding cheetahs and considering life history of individual cheetahs. Of the factors considered, we only found three main husbandry aspects that correlated with cheetah MSIs: nutrition, management of pregnant females, and enrichment type. Specifically, inclusion of ruminant meats in female cheetah diets resulted in larger litter sizes, and females who experienced starve days outside of the breeding season had greater proportion of stillbirths. Similarly, the proportion of stillbirths was also greater in litters of females who were moved to a new enclosure for pregnancy and parenting, which was also associated with an increased reported risk of maternal negligence. Finally, while enrichment provision overall had no effect on female cheetah reproductive success, two enrichment types appeared to negatively impact success: respondents reported higher risk of negligence on females provided with catnip, and the use of feederballs was associated with less cubs mothered to 6 weeks and beyond. Possible reasons and implications of these findings are discussed in turn below. A high number of factors were tested in this study following initial study. These are discussed below, as well as limitations around interpretation (given the small sample sizes and overall exploratory nature of this study), as well as ideas for future focused, experimental studies.

### 4.1 Life‐History Factors

Age and maternal experience were uncorrelated to maternal success. This is consistent with one small‐sample study of wild cheetah females in the Serengeti (Laurenson 1993) but contradicts a more recent study suggesting recruitment in wild female cheetahs peaks at 6–7 years old (Durant 2004). As lion predation is a leading cause of wild cheetah cub mortality (Durant 2004; Laurenson et al. 1995), the removal of predation in an ex situ environment may explain the lack of relationship between age and parity in our study, as maternal experience surrounding predator avoidance is not a requirement for captive success. Consistent with this, we found that increased number of generations in captivity—assessed via matriline—tend to correlate with reduced occurrence of stillbirths. It is also possible that given the mild heritability of maternal success (Bertschinger et al. 2008), this trend may reflect inadvertent selection of successful mothers over time and a larger sample size could produce a different statistical outcome. Similarly, captive breeding might unconsciously select for tamer, less stressed cheetahs. While cheetahs are not part of any taming experiments, extensive research on foxes (*Vulpes vulpes*) has shown deliberately selecting for tameness results in friendlier behaviour in six generations and that stress hormone levels have halved in 15 generations when compared to untamed foxes (Dugatkin 2018; Trut 1999). This is relevant because elevation in stress is a risk factor for stillbirth (see below). Cheetahs displaying phenotypic “stressed” behaviour may also be less successful parents: research suggests that captive cheetahs described as “fearful‐tense” are less reproductively successful than other behaviour types, such as “excitable‐vocal” (Wielebnowski 1999). This can be related to physiological stress responses: a recent study in cheetahs has shown that fecal glucocorticoid levels in mothers of single cub litters is higher than those of mothers of multi‐cub litters so it is plausible that stress influences maternal success from conception (Koester et al. 2022). A focused study with a larger sample size comparing a cheetah female's cub production compared with her ex situ ancestry would be the next step to better investigate this apparent tendency.

### 4.2 Husbandry: The Effects of Nutrition on Reproductive Success

Two aspects of nutrition appeared to have an effect on reproductive success. First, feeding of ruminant meat and carcasses was found to be significantly related to a larger average litter size. Wild female cheetahs frequently consume small antelope species (Marker et al. 2003) and so the inclusion of this meat in the diet of ex situ cheetahs better mimics a wild diet. An explanation for the breeding relevance of this may lie in fatty acids. Ruminant meat typically has a lower fatty acid saturated to unsaturated fat ratio than monogastric meats, and wild Namibian cheetahs have been found to have lower blood serum fatty acid levels but higher arachidonic acid levels than captive cheetahs fed commercially (Tordiffe et al. 2016). Fatty acids are also linked with breeding in felines: captive acyclic cheetahs with low fatty acid levels have been noted to restart cycling following fatty acid supplementation (Davidson et al. 1986). In domestic cats, queens (females) are unable to breed without sufficient poly‐unsaturated fatty acids and arachidonic acid (Morris 2004). Therefore, it is plausible that feeding ruminant meat ex situ may result in levels of fatty acid closer to the wild norm, resulting in larger litter sizes, although further research would be needed to confirm this is a causal relationship.

Second, fasting through provision of starve days did not improve any MSIs and instead correlated with an increase percentage of stillbirths per litter. Starve days in captivity are practiced under the assumption that they mimic wild feeding patterns in large felids and, consequently, cater better to the natural biology of the animal, though ARBs are known to be reduced in cheetahs with regular, predictable feeding patterns (Quirke et al. 2012). Starve days have been shown to increase activity while decreasing ARBs in lions (*Panthera leo*), which is considered beneficial and has been speculated to also apply to cheetahs (Altman et al. 2005; Sengenberger et al. 2018). In contrast, Amur tigers (*Panthera tigris altaica*), jaguars (*Panthera onca*), Persian leopards (*Panthera pardus saxicolor*) and snow leopards (*Panthera uncia*) exposed to regular starve days display increased levels of ARBs on starve days than on feed days (Lyons et al. 1997). One zoo manual recommends against using starve days for breeding animals, citing a potential for causing animals stress as a disadvantage to this practice (McLeod 1999). A starve day is not the same as a wild fast‐gorge feeding pattern because the actions available to wild cheetahs that could be taken to change a fast situation (e.g., increasing hunting efforts, moving to a new area) are inaccessible to captive cheetahs, and so frustration or stress could feasibly be caused (Sengenberger et al. 2018), with knock‐on undesirable effects for reproduction. Stress has been linked to stillbirth in humans and dogs (*Canis familiaris*) (Bennett 1974; Sengenberger et al. 2018; Silver et al. 2007) and elevated glucocorticoids in cheetahs is associated with smaller litters (Koester et al. 2022).

It is plausible that zoos practicing starve days also practice other husbandry techniques or have another common factor that is uncaptured by this study, meaning that starve days are confounding or interacting with other potential influencing factors. Further experimental research should be conducted to ascertain causality. It would be particularly useful to know the timeframes and frequency of feeding practices, though this can be difficult to capture on a multi‐institution level.

### 4.3 Husbandry: Enrichment and Reproductive Success

When all enrichment practices were grouped together, general enrichment provision did not appear to result in a more positive mothering outcome. This contradicts suggestion that insufficient enrichment in Amur tigers could result in compromised reproductive success (Szokalski et al. 2012) and findings in giant pandas where enrichment has been noted as a method of promoting reproductive behaviour (Maple and Perdue 2013) but matches findings in American mink that indicate enrichment does not impact pre or post copulatory behaviour in females nor on the growth of their offspring (Díez‐León and Mason, 2016; Díez‐León 2014). However, two types of enrichments—one diet related, one sensory—unexpectedly had negative effects on maternal success when examined separately. The provision of feederballs used by females when not actively pregnant or with cubs was associated with a lower rate of STMS and LTMS (NB. there was not enough data to test effects of feederball provision during pregnancy). Limited literature is published surrounding feederballs in cheetahs, suggesting that their use may be based on practical trial‐and‐error, habit or tradition rather than species‐specific research (Ahlrot 2016). In domestic cats, feederball use positively impacts welfare, though the effects on mothering are unknown (Dantas et al. 2016), but this may be due to differing feeding patterns between cats and cheetahs. While cats have evolved to catch multiple small meals within a day, cheetahs have evolved to feed on a gorge‐fast cycle, so it is possible that feederballs themselves do not compliment the natural biology of the cheetah (Bradshaw 2006; Sengenberger et al. 2018). It is unclear whether our result truly reflects effects of this enrichment item, more general alterations in a female's usual enrichment protocol or even usage. The provision of an enrichment device does not necessarily equate to use by the animal and there is no evidence to support that using a feederball during the life of a cheetah should directly biologically impact that cheetah's maternal psychology. Our result might be erroneous (i.e., a Type I error due to multiple testing). Future work should seek to collect data on feederball use by female cheetahs to fully elucidate welfare and reproductive effects of feederballs. In the meantime, consideration of wild feeding biology should continue to inform enrichment choices in zoological collections.

Cheetahs who were enriched with catnip as part of their enrichment schedule were more likely to be reported as negligent by keepers, though it is important to note that this study did not capture how close this enrichment was used to parenting. The physical effects of catnip on domestic cats are mediated through olfaction (Hart and Leedy 1985). Few studies have investigated health and safety in regards to olfactory stimulation, including by catnip, in zoo animals (Clark and King 2008); to our knowledge, there are also no studies investigating the pharmacological effects of catnip in mammals. Catnip is used within zoos for cheetah enrichment and has been studied as an enrichment device but has limited scientific studies on effects in recreational feline users (Clark and King 2008; Damasceno et al. 2017). While maternal success in association with catnip has not previously been researched, there is a known association between catnip effects and sexual maturity. Sexually mature lions and leopards have been noted to be more behaviourally reactive to catnip than juveniles or geriatrics (Hill et al. 1976), though in domestic cats, both neutered and unneutered animals may respond to catnip (Grognet 1990; Hill et al. 1976). As with our feederball result, without further studies, it is unclear whether the link between increased negligence rate and catnip use in cheetahs is causal or spurious.

### 4.4 Husbandry: Effects of Moving Breeding Females and Reproductive Success

Mate choice was found to have no effect on mothering, experimental findings in giant pandas, where mate choice results in increased cub production and more successful cub raising (Martin‐Wintle et al. 2015). This is also contrary to findings in non‐carnivora mammals as mate choice has been found to increase litter sizes and offspring outcomes in house mice (*Mus musculus*) and Columbia basin pygmy rabbits (*Brachylagus idahoenis*) (Drickamer et al. 2000; Martin and Shepherdson 2012). The sample population for this study was females who had mated, which influences the finding for this variable as it does not capture females who had rejected a male and who consequently had not mated.

Moving to a new enclosure for pregnancy and parenting resulted in a higher proportion of stillbirths and reported negligence. As the motivation for zoos moving an expectant mother is unknown, it is possible that this result reflects females judged high‐risk being more frequently moved. EAZA guidelines recommend camera‐monitoring for parturition and while these standards would be something our subject zoos are working towards, the questionnaire did not capture if this was the case: it may be that some females were moved for practical reasons in order for zoos to utilise monitoring access and equipment (Sengenberger et al. 2018). Regardless of rationale, the stress of an enclosure move should not be discounted. Previous research has described elevated fecal corticoids for up to 60 days in several zoo‐housed felid species, including cheetahs, when moved to new exhibits (Moreira et al. 2007; Wells et al. 2004). In tigrinas (*L. tigrinus*) and margays (*L. wiedii*), the movement of females from large enriched enclosures to smaller, barren enclosures resulted in agitation, and stereotypic pacing (Moreira et al. 2007). Alteration in enclosure husbandry similarly leads to chronic stress (Carlstead et al. 1993). A link between enclosure disruption and maternal behaviour is unstudied in felids but in rats, increased cage disruption frequency correlates with decreased maternal success (Burn and Mason 2008). While stillbirth is not fully understood in any species, stress has been linked to dystocia and stillbirth (see above) (Bennett 1974; Silver et al. 2007). A behavioural study would be needed to formally investigate this survey‐based effect.

## 5 Conclusions

The findings of this study suggest that maternal success in cheetahs could potentially be increased through the feeding of ruminant meat, the elimination of the practice of starve days, and by avoiding enclosure moves for pregnant and mothering cheetahs. While enrichment itself was found to have no effect on maternal success, the use of feederballs and catnip as enrichment devices were associated with poorer mothering outcomes. Our results consequently highlighted an inconsistency in published evidence versus practiced animal husbandry, particularly regarding the practice of starve days and provision of two types of enrichment. Evidence‐based animal management in ex situ collections is required not only in enrichment in breeding cheetahs but in all aspects of care in all species. Without this, the validity and ethics of collection animals becomes questionable. Further experimental investigation into animal enrichment devices, feeding, accommodation and stress in relation to maternal success in cheetahs is needed to better understand what truly makes a mother.

## Ethic Statement

The authors have nothing to report.

## Conflicts of Interest

The authors declare no conflicts of interest.

## Supporting information

Supporting information.

## Data Availability

Anonymised data that supports the findings in this paper is available upon request from author Sian Barr.

## References

[zoo21894-bib-0001] Ahlrot, U. 2016. “Applied Ethology And Animal Biology.” In Welfare in Zoo Kept Felids: A Study of Resource Usage. Linköping University.

[zoo21894-bib-0002] Altman, J. D. , K. L. Gross , and S. R. Lowry . 2005. “Nutritional and Behavioral Effects of Gorge and Fast Feeding in Captive Lions.” Journal of Applied Animal Welfare Science 8: 47–57. 10.1207/s15327604jaws0801_4. 16004544

[zoo21894-bib-0003] Alves, S. E. , P. H. Joyner , C. Aitken‐Palmer , A. E. Crosier , and L. Ware . 2018. “Full‐Term Pregnancy With Vaginal Birth Following Dystocia and Caesarean Section in Two Cheetahs (*Acinonyx jubatus*).” Veterinary Record Case Reports 6: e000582. 10.1136/vetreccr-2017-000582.

[zoo21894-bib-0004] Asa, C. S. , K. Traylor‐Holzer , and R. C. Lacy . 2011. “Can Conservation‐Breeding Programmes be Improved by Incorporating Mate Choice?” International Zoo Yearbook 45: 203–212. 10.1111/j.1748-1090.2010.00123.x.

[zoo21894-bib-0005] Augustus, P. , K. Casavant , N. Troxel , R. Rieches , and F. Bercovitch . 2006. “Reproductive Life History of South African Cheetahs (*Acynonyx Jubatus Jubatus*) at the San Diego Zoo Wild Animal Park, 1970–2005.” Zoo Biology 25: 383–390. 10.1002/zoo.20097.

[zoo21894-bib-0006] Balme, G. A. , A. Batchelor , N. de Woronin Britz , et al. 2013. “Reproductive Success of Female Leopardspanthera Pardus: The Importance of Top‐Down Processes.” Mammal Review 43: 221–237. 10.1111/j.1365-2907.2012.00219.x.

[zoo21894-bib-0007] Baruch, Y. , and B. C. Holtom . 2008. “Survey Response Rate Levels and Trends in Organizational Research.” Human Relations 61: 1139–1160. 10.1177/0018726708094863.

[zoo21894-bib-0008] Beekman, S. P. A. , B. Kemp , H. C. M. Louwman , and B. Colenbrander . 1999. “Analyses of Factors Influencing the Birth Weight and Neonatal Growth Rate of Cheetah (*Acinonyx jubatus*) Cubs.” Zoo Biology 18: 129–139. 10.1002/(SICI)1098-2361(1999)18:2<129::AID-ZOO4>3.0.CO;2-9.

[zoo21894-bib-0009] Bennett, D. 1974. “Canine Dystocia‐‐A Review of the Literature.” Journal of Small Animal Practice 15: 101–117. 10.1111/j.1748-5827.1974.tb05667.x. 4615208

[zoo21894-bib-0010] Bertschinger, H. , D. Meltzer , and A. van Dyk . 2008. “Captive Breeding of Cheetahs in South Africa: 30 Years of Data From the De Wildt Cheetah and Wildlife Centre.” Reproduction in Domestic Animals 43: 66–73. 10.1111/j.1439-0531.2008.01144.x. 18638106

[zoo21894-bib-0011] Bradshaw, J. W. S. 2006. “The Evolutionary Basis for the Feeding Behavior of Domestic Dogs (Canis Familiaris) and Cats (*Felis catus*).” Journal of Nutrition 136: 1927S–1931S. 10.1093/jn/136.7.1927S. 16772461

[zoo21894-bib-0012] Burn, C. C. , and G. J. Mason . 2008. “Effects of Cage‐Cleaning Frequency on Laboratory Rat Reproduction, Cannibalism, and Welfare.” Applied Animal Behaviour Science 114: 235–247. 10.1016/j.applanim.2008.02.005.

[zoo21894-bib-0013] Carlstead, K. , J. L. Brown , and W. Strawn . 1993. “Behavioral and Physiological Correlates of Stress in Laboratory Cats.” Applied Animal Behaviour Science 38: 143–158. 10.1016/0168-1591(93)90062-T.

[zoo21894-bib-0014] Caro, T. M. 1987. “Cheetah Mothers’ Vigilance: Looking Out for Prey or for Predators?” Behavioral Ecology and Sociobiology 20: 351–361. 10.1007/BF00300681.

[zoo21894-bib-0015] Charruau, P. , C. Fernandes , P. Orozco‐Terwengel , et al. 2011. “Phylogeography, Genetic Structure and Population Divergence Time of Cheetahs in Africa and Asia: Evidence for Long‐Term Geographic Isolates.” Molecular Ecology 20: 706–724. 10.1111/j.1365-294X.2010.04986.x. 21214655 PMC3531615

[zoo21894-bib-0016] Clark, F. , and A. J. King . 2008. “A Critical Review of Zoo‐Based Olfactory Enrichment.” In Chemical Signals in Vertebrates 11, edited by J. L. Hurst , R. J. Beynon , S. C. Roberts , and T. D. Wyatt , 391–398. 10.1007/978-0-387-73945-8_37.

[zoo21894-bib-0017] Clubb, R. , and G. J. Mason . 2007. “Natural Behavioural Biology as a Risk Factor in Carnivore Welfare: How Analysing Species Differences Could Help Zoos Improve Enclosures.” Applied Animal Behaviour Science 102: 303–328. 10.1016/j.applanim.2006.05.033.

[zoo21894-bib-0018] Connor, K. L. , M. H. Vickers , J. Beltrand , M. J. Meaney , and D. M. Sloboda . 2012. “Nature, Nurture or Nutrition? Impact of Maternal Nutrition on Maternal Care, Offspring Development and Reproductive Function.” The Journal of Physiology 590: 2167–2180. 10.1113/jphysiol.2011.223305. 22411006 PMC3447158

[zoo21894-bib-0019] Conway, W. G. 2010. “Buying Time for Wild Animals With Zoos.” Zoo Biology 30: 1–8. 10.1002/zoo.20352. 20938970

[zoo21894-bib-0020] Crosier, A. E. , P. Comizzoli , T. Baker , et al. 2011. “Increasing Age Influences Uterine Integrity, But Not Ovarian Function or Oocyte Quality, in the Cheetah (*Acinonyx jubatus*)1.” Biology of Reproduction 85: 243–253. 10.1095/biolreprod.110.089417. 21565998

[zoo21894-bib-0021] Cutuli, D. , P. Caporali , F. Gelfo , et al. 2015. “Pre‐Reproductive Maternal Enrichment Influences Rat Maternal Care and Offspring Developmental Trajectories: Behavioral Performances and Neuroplasticity Correlates.” Frontiers in Behavioral Neuroscience 9: 66. 10.3389/fnbeh.2015.00066. 25814946 PMC4357301

[zoo21894-bib-0022] De Cuyper, A. , M. Clauss , C. Carbone , et al. 2019. “Predator Size and Prey Size–Gut Capacity Ratios Determine Kill Frequency and Carcass Production in Terrestrial Carnivorous Mammals.” Oikos 128: 13–22. 10.1111/oik.05488.

[zoo21894-bib-0023] Damasceno, J. , G. Genaro , T. Quirke , S. McCarthy , S. McKeown , and R. O'Riordan . 2017. “The Effects of Intrinsic Enrichment on Captive Felids.” Zoo Biology 36: 186–192. 10.1002/zoo.21361. 29165868

[zoo21894-bib-0024] Dantas, L. M. , M. M. Delgado , I. Johnson , and C. T. Buffington . 2016. “Food Puzzles for Cats: Feeding for Physical and Emotional Wellbeing.” Journal of Feline Medicine and Surgery 18: 723–732. 10.1177/1098612X16643753. 27102691 PMC11148901

[zoo21894-bib-0025] Davidson, B. C. , R. C. Cantrill , and D. Varaday . 1986. “The Reversal of Essential Fatty Acid Deficiency Symptoms in the Cheetah.” South African Journal of Zoology 21: 161–164. 10.1080/02541858.1986.11447974.

[zoo21894-bib-0026] Díez‐León, M. 2014. Effects of Environmental Enrichment on Stereotypic Behaviour and Reproductive Success in American mink *Neovison vison* . University of Guelph. 10.1002/zoo.21249 26536278

[zoo21894-bib-0027] Díez‐León, M. , and G. Mason . 2016. “Effects of Environmental Enrichment and Stereotypic Behavior on Maternal Behavior and Infant Viability in a Model Carnivore, the American Mink (*Neovison vison*): Enrichment Effects on Maternal Behavior.” Zoo Biology 35: 19–28. 10.1002/zoo.21249. 26536278

[zoo21894-bib-0028] Drickamer, L. C. , P. A. Gowaty , and C. M. Holmes . 2000. “Free Female Mate Choice in House Mice Affects Reproductive Success and Offspring Viability and Performance.” Animal Behaviour 59: 371–378. 10.1006/anbe.1999.1316. 10675259

[zoo21894-bib-0029] Dugatkin, L. A. 2018. “The Silver Fox Domestication Experiment.” Evolution: Education and Outreach 11: 16. 10.1186/s12052-018-0090-x.

[zoo21894-bib-0030] Durant, S. M. 2000. “Predator Avoidance, Breeding Experience and Reproductive Success in Endangered Cheetahs, *Acinonyx jubatus* .” Animal Behaviour 60: 121–130. 10.1006/anbe.2000.1433. 10924211

[zoo21894-bib-0031] Durant, S. M. 2004. “Factors Affecting Life and Death in Serengeti Cheetahs: Environment, Age, and Sociality.” Behavioral Ecology 15: 11–22. 10.1093/beheco/arg098.

[zoo21894-bib-0032] Fazio, J. M. , E. W. Freeman , E. Bauer , L. Rockwood , and E. C. M. Parsons . 2019. “Evaluation of Management in North American Zoos to Enhance Breeding Success of the Fishing Cat (*Prionailurus viverrinus*) Ex Situ Population.” Zoo Biology 38: 189–199. 10.1002/zoo.21465. 30556919

[zoo21894-bib-0033] Folio, D. M. , J. Aars , O. Gimenez , A. E. Derocher , Ø. Wiig , and S. Cubaynes . 2019. “How Many Cubs Can a Mum Nurse? Maternal Age and Size Influence Litter Size in Polar Bears.” Biology Letters 15: 20190070. 10.1098/rsbl.2019.0070. 31039729 PMC6548740

[zoo21894-bib-0034] Foreman, G. E. 1997. “Breeding and Maternal Behaviour in Geoffroy's Cats Oncifelis Geoffroyi.” International Zoo Yearbook 35: 104–115. 10.1111/j.1748-1090.1997.tb01198.x.

[zoo21894-bib-0035] Forti, I. R. N. 2019. What Studbooks Can Tell Us About Captive Breeding Programmes: A Case Study of Cheetahs (*Acinonyx jubatus*) (PhD Thesis). University Of Salford Manchester.

[zoo21894-bib-0036] Fox, J. , C. Murray , and A. Warm . 2003. “Conducting Research Using Web‐Based Questionnaires: Practical, Methodological, and Ethical Considerations.” International Journal of Social Research Methodology 6: 167–180. 10.1080/13645570210142883.

[zoo21894-bib-0037] Grognet, J. 1990. “Catnip: Its Uses and Effects, Past and Present.” Canadian Veterinary Journal = La revue veterinaire canadienne 31: 455–456. 17423611 PMC1480656

[zoo21894-bib-0038] Hampson, M. C. , and C. Schwitzer . 2016. “Effects of Hand‐Rearing on Reproductive Success in Captive Large Cats *Panthera Tigris* Altaica, *Uncia Uncia*, *Acinonyx jubatus* and *Neofelis Nebulosa* .” PLoS One 11: e0155992. 10.1371/journal.pone.0155992. 27214261 PMC4877043

[zoo21894-bib-0039] Hart, B. L. , and M. G. Leedy . 1985. “Analysis of the Catnip Reaction: Mediation by Olfactory System, Not Vomeronasal Organ.” Behavioral and Neural Biology 44: 38–46. 10.1016/S0163-1047(85)91151-3. 3834921

[zoo21894-bib-0040] Hill, J. O. , E. J. Pavlik , G. L. Smith , G. M. Burghardt , and P. B. Coulson . 1976. “Species‐Characteristic Responses to Catnip by Undomesticated Felids.” Journal of Chemical Ecology 2: 239–253. 10.1007/BF00987747.

[zoo21894-bib-0041] Kelly, M. J. 2001. “Lineage Loss in Serengeti Cheetahs: Consequences of High Reproductive Variance and Heritability of Fitness on Effective Population Size.” Conservation Biology 15: 137–147. 10.1111/j.1523-1739.2001.99033.x.

[zoo21894-bib-0042] Kelly, M. J. , M. K. Laurenson , C. D. Fitzgibbon , et al. 1998. “Demography of the Serengeti Cheetah (*Acinonyx jubatus*) Population: The First 25 Years.” Journal of Zoology 244: 473–488. 10.1111/j.1469-7998.1998.tb00053.x.

[zoo21894-bib-0043] Koester, D. , M. Maly , S. Putman , K. Edwards , K. Meeks , and A. Crosier . 2022. “An Investigation of Ovarian and Adrenal Hormone Activity in Post‐Ovulatory Cheetahs (*Acinonyx jubatus*).” Animals: An Open Access Journal From MDPI 12: 809. 10.3390/ani12070809. 35405799 PMC8996957

[zoo21894-bib-0044] Lacy, R. C. 2013. “Achieving True Sustainability of Zoo Populations.” Zoo Biology 32: 19–26. 10.1002/zoo.21029. 22753040

[zoo21894-bib-0045] Latham, N. R. , and G. J. Mason . 2008. “Maternal Deprivation and the Development of Stereotypic Behaviour.” Applied Animal Behaviour Science 110: 84–108. 10.1016/j.applanim.2007.03.026.

[zoo21894-bib-0046] Laurenson, M. K. 1993. “Early Maternal Behavior of Wild Cheetahs: Implications for Captive Husbandry.” Zoo Biology 12: 31–43. 10.1002/zoo.1430120106.

[zoo21894-bib-0047] Laurenson, M. K. 1994. “High Juvenile Mortality in Cheetahs (*Acinonyx jubatus*) and Its Consequences for Maternal Care.” Journal of Zoology 234: 387–408. 10.1111/j.1469-7998.1994.tb04855.x.

[zoo21894-bib-0048] Laurenson, M. K. , T. M. Caro , and M. Borner . 1992. “Female Cheetah Reproduction.” National Geographic Research & Exploration 8: 64–75.

[zoo21894-bib-0049] Laurenson, M. K. , N. Wielebnowski , and T. M. Caro . 1995. “Extrinsic Factors and Juvenile Mortality in Cheetahs.” Conservation Biology 9: 1329–1331. 34261268 10.1046/j.1523-1739.1995.9051327.x-i1

[zoo21894-bib-0050] Lees, C. M. , and J. Wilcken . 2009. “Sustaining the Ark: The Challenges Faced by Zoos in Maintaining Viable Populations.” International Zoo Yearbook 43: 6–18. 10.1111/j.1748-1090.2008.00066.x.

[zoo21894-bib-0051] Little, H. A. , T. C. Gilbert , M. L. Athorn , and A. R. Marshall . 2016. “Evaluating Conservation Breeding Success for an Extinct‐In‐The‐Wild Antelope.” PLoS One 11: e0166912. 10.1371/journal.pone.0166912. 27935999 PMC5147836

[zoo21894-bib-0052] Liu, D. , J. Diorio , J. C. Day , D. D. Francis , and M. J. Meaney . 2000. “Maternal Care, Hippocampal Synaptogenesis and Cognitive Development in Rats.” Nature Neuroscience 3: 799–806. 10.1038/77702. 10903573

[zoo21894-bib-0053] Loberg, J. M. , M. Slof Pacilio , L. Lundin , and E. Andersson . 2020. “Survey to Identify Factors Affecting Breeding of Wolverines *Gulo gulo* Within the Eep.” International Zoo Yearbook 54: 86–101. 10.1111/izy.12269.

[zoo21894-bib-0054] Lyons, J. , R. J. Young , and J. M. Deag . 1997. “The Effects of Physical Characteristics of the Environment and Feeding Regime on the Behavior of Captive Felids.” Zoo Biology 16: 71–83. 10.1002/(SICI)1098-2361(1997)16:1<71::AID-ZOO8>3.0.CO;2-8.

[zoo21894-bib-0055] Maple, T. L. , and B. M. Perdue . 2013. “Environmental Enrichment.” In Zoo Animal Welfare, Animal Welfare, edited by T. Maple and B. M. Perdue , 95–117. Springer. 10.1007/978-3-642-35955-2_6.

[zoo21894-bib-0056] Marker, L. , M. Gusset , K. Vannelli , and L. Versteege . 2018. “History of Cheetahs in Zoos and Demographic Trends Through Managed Captive Breeding Programs.” In Cheetahs: Biology and Conservation, 309–321. Academic Press. 10.1016/B978-0-12-804088-1.00022-8.

[zoo21894-bib-0057] Marker, L. , and S. J. O'Brien . 1989. “Captive Breeding of the Cheetah (*Acinonyx jubatus*) in North American Zoos (1871–1986).” Zoo Biology 8: 3–16. 10.1002/zoo.1430120104.

[zoo21894-bib-0058] Marker, L. L. , J. R. Muntifering , A. J. Dickman , M. Mills , and D. Macdonald . 2003. “Quantifying Prey Preferences of Free‐Ranging Namibian Cheetahs.” African Journal of Wildlife Research 33: 43–53.

[zoo21894-bib-0059] Martin, M. S. , and D. J. Shepherdson . 2012. “Role of Familiarity and Preference in Reproductive Success in Ex Situ Breeding Programs.” Conservation Biology 26: 649–656. 10.1111/j.1523-1739.2012.01880.x. 22809353

[zoo21894-bib-0060] Martin‐Wintle, M. S. , D. Shepherdson , G. Zhang , et al. 2015. “Free Mate Choice Enhances Conservation Breeding in the Endangered Giant Panda.” Nature Communications 6: 10125. 10.1038/ncomms10125. PMC4682106 26670381

[zoo21894-bib-0061] Martin‐Wintle, M. S. , N. J. P. Wintle , M. Díez‐León , R. R. Swaisgood , and C. S. Asa . 2019. “Improving the Sustainability of Ex Situ Populations With Mate Choice.” Zoo Biology 38: 119–132. 10.1002/zoo.21450. 30474268

[zoo21894-bib-0062] McLeod, G. 1999. The Environmental Husbandry Manual ‐ Individual Methods ‐ Starve Days [WWW Document]. Wildpro. http://wildpro.twycrosszoo.org/S/00Ref/miscellaneouscontents/20_FeedingPractises/StarveDays.htm.

[zoo21894-bib-0063] Meagher, R. K. , J. Ahloy Dallaire , D. L. M. Campbell , et al. 2014. “Benefits of a Ball and Chain: Simple Environmental Enrichments Improve Welfare and Reproductive Success in Farmed American Mink (*Neovison vison*).” PLoS One 9: e110589. 10.1371/journal.pone.0110589. 25386726 PMC4227648

[zoo21894-bib-0064] Mellen, J. D. 1991. “Factors Influencing Reproductive Success in Small Captive Exotic Felids (Felis Spp.): A Multiple Regression Analysis.” Zoo Biology 10: 95–110. 10.1002/zoo.1430100202.

[zoo21894-bib-0065] Mellen, J. D. 1992. “Effects of Early Rearing Experience on Subsequent Adult Sexual Behavior Using Domestic Cats (*Felis catus*) as a Model for Exotic Small Felids.” Zoo Biology 11: 17–32. 10.1002/zoo.1430110104.

[zoo21894-bib-0066] Mellen, J. D. , and D. J. Shepherdson . 1997. “Environmental Enrichment for Felids: An Integrated Approach.” International Zoo Yearbook 35: 191–197. 10.1111/j.1748-1090.1997.tb01209.x.

[zoo21894-bib-0067] Moreira, N. , J. L. Brown , W. Moraes , W. F. Swanson , and E. L. A. Monteiro‐Filho . 2007. “Effect of Housing and Environmental Enrichment on Adrenocortical Activity, Behavior and Reproductive Cyclicity in the Female Tigrina (*Leopardus Tigrinus*) and Margay (*Leopardus Wiedii*).” Zoo Biology 26: 441–460. 10.1002/zoo.20139. 19360593

[zoo21894-bib-0068] Morris, J. G. 2004. “Do Cats Need Arachidonic Acid in the Diet for Reproduction?” Journal of Animal Physiology and Animal Nutrition 88: 131–137. 10.1111/j.1439-0396.2003.00469.x. 15059237

[zoo21894-bib-0069] Mossotti, R. H. 2010. Female Reaction to Male Urine Scents as Potential Indicator of Mate Choice in Captive Cheetahs (*Acinonyx jubatus*). Southern Illinois University Carbondale.

[zoo21894-bib-0070] Mossotti, R. H. , E. A. Baskir , C. P. Kozlowski , A. D. Franklin , G. A. Feldhamer , and C. S. Asa . 2018. “Reactions of Female Cheetahs (*Acinonyx jubatus*) to Urine Volatiles From Males of Varying Genetic Distance.” Zoo Biology 37: 229–235. 10.1002/zoo.21420. 29900582

[zoo21894-bib-0071] Newberry, R. C. 1995. “Environmental Enrichment: Increasing the Biological Relevance of Captive Environments.” Applied Animal Behaviour Science 44: 229–243. 10.1016/0168-1591(95)00616-Z.

[zoo21894-bib-0072] Pekár, S. , and M. Brabec . 2018. “Generalized Estimating Equations: A Pragmatic and Flexible Approach to the Marginal GLM Modelling of Correlated Data in the Behavioural Sciences.” Ethology 124: 86–93. 10.1111/eth.12713.

[zoo21894-bib-0073] Quirke, T. , and R. O'Riordan . 2015. “An Investigation Into the Prevalence of Exploratory Behavior in Captive Cheetahs (*Acinonyx jubatus*).” Zoo Biology 34: 130–138. 10.1002/zoo.21193. 25557735

[zoo21894-bib-0074] Quirke, T. , and R. M. O'Riordan . 2011. “The Effect of a Randomised Enrichment Treatment Schedule on the Behaviour of Cheetahs (*Acinonyx jubatus*).” Applied Animal Behaviour Science 135: 103–109. 10.1016/j.applanim.2011.10.006.

[zoo21894-bib-0075] Quirke, T. , R. M. O'Riordan , and A. Zuur . 2012. “Factors Influencing the Prevalence of Stereotypical Behaviour in Captive Cheetahs (*Acinonyx jubatus*).” Applied Animal Behaviour Science 142: 189–197. 10.1016/j.applanim.2012.09.007.

[zoo21894-bib-0076] Schou, T. M. , R. Palme , and J. Malmkvist . 2019. “Relocation Shortly after Mating Does Not Have a Major Impact on Stress Responses and Reproduction in Female Farm Mink.” Applied Animal Behaviour Science 214: 89–94. 10.1016/j.applanim.2019.03.007.

[zoo21894-bib-0077] Sengenberger, K. , H. Bus , and L. Versteege , 2018. EAZA Best Practice Guidelines Cheetah (*Acinonyx jubatus*) 147.

[zoo21894-bib-0078] Setchell, K. D. R. , S. J. Gosselin , M. B. Welsh , et al. 1987. “Dietary Estrogens—A Probable Cause of Infertility and Liver Disease in Captive Cheetahs.” Gastroenterology 93: 225–233. 10.5555/uri:pii:0016508587910067. 3297906

[zoo21894-bib-0079] Shepherdson, D. J. , J. D. Mellen , and M. Hutchins . 2012. Second Nature: Environmental Enrichment for Captive Animals. Smithsonian Institution.

[zoo21894-bib-0080] Siciliano‐Martina, L. 2020. “Multi‐Institutional Survey of Causes of Maternal Rejection in Giraffes *Giraffa Camelopardalis* in North American Zoos.” International Zoo Yearbook 54: 191–201. 10.1111/izy.12252.

[zoo21894-bib-0081] Silver, R. M. , M. W. Varner , U. Reddy , et al. 2007. “Work‐Up of Stillbirth: A Review of the Evidence.” American Journal of Obstetrics and Gynecology 196: 433–444. 10.1016/j.ajog.2006.11.041. 17466694 PMC2699761

[zoo21894-bib-0082] Sunquist, M. , and F. Sunquist . 2017. Wild Cats of the World. University of Chicago Press.

[zoo21894-bib-0083] SurveyMonkey . 2019. SurveyMonkey [WWW Document]. SurveyMonkey: The UK's Most Popular Free Online Survey Tool. https://www.surveymonkey.co.uk/ (accessed 8.14.19).

[zoo21894-bib-0084] Szokalski, M. S. , C. A. Litchfield , and W. K. Foster . 2012. “Enrichment for Captive Tigers (*Panthera tigris*): Current Knowledge and Future Directions.” Applied Animal Behaviour Science 139: 1–9. 10.1016/j.applanim.2012.02.021.

[zoo21894-bib-0085] Tanaka, A. , and T. Ogura . 2018. “Current Husbandry Situation of Red Pandas in Japan.” Zoo Biology 37: 107–114. 10.1002/zoo.21407. 29512188

[zoo21894-bib-0086] Tidière, M. , P. Müller , A. Sliwa , A. Siberchicot , and G. Douay . 2021. “Sex‐Specific Actuarial and Reproductive Senescence in Zoo‐Housed Tiger (*Panthera tigris*): The Importance of Sub‐Species for Conservation.” Zoo Biology 40: 320–329. 10.1002/zoo.21610. 33861886

[zoo21894-bib-0087] Tordiffe, A. S. W. , B. Wachter , S. K. Heinrich , F. Reyers , and L. J. Mienie . 2016. “Comparative Serum Fatty Acid Profiles of Captive and Free‐Ranging Cheetahs (*Acinonyx jubatus*) in Namibia.” PLoS One 11: e0167608. 10.1371/journal.pone.0167608. 27992457 PMC5167222

[zoo21894-bib-0088] Trut, L. 1999. “Early Canid Domestication: The Farm‐Fox Experiment.” American Scientist 87: 160–169.

[zoo21894-bib-0089] Wells, A. , K. A. Terio , M. H. Ziccardi , and L. Munson . 2004. “THE Stress Response To Environmental Change IN Captive Cheetahs (*Acinonyx jubatus*).” Journal of Zoo and Wildlife Medicine 35: 8–14. 10.1638/02-084. 15193067

[zoo21894-bib-0090] Whitehouse‐Tedd, K. M. , S. L. Lefebvre , and G. P. J. Janssens . 2015. “Dietary Factors Associated With Faecal Consistency and Other Indicators of Gastrointestinal Health in the Captive Cheetah (*Acinonyx jubatus*).” PLoS One 10: e0120903. 10.1371/journal.pone.0120903. 25830636 PMC4382097

[zoo21894-bib-0091] Wielebnowski, N. 1996. “Reassessing the Relationship Between Juvenile Mortality and Genetic Monomorphism in Captive Cheetahs.” Zoo Biology 15: 353–369. 10.1002/(SICI)1098-2361(1996)15:4<353::AID-ZOO1>3.0.CO;2-A.

[zoo21894-bib-0092] Wielebnowski, N. C. 1999. “Behavioral Differences as Predictors of Breeding Status in Captive Cheetahs.” Zoo Biology 18: 335–349. 10.1002/(SICI)1098-2361(1999)18:4<335::AID-ZOO8>3.0.CO;2-X.

[zoo21894-bib-0093] Willoughby, J. R. , J. A. Ivy , R. C. Lacy , J. M. Doyle , and J. A. DeWoody . 2017. “Inbreeding and Selection Shape Genomic Diversity in Captive Populations: Implications for the Conservation of Endangered Species.” PLoS One 12: e0175996. 10.1371/journal.pone.0175996. 28423000 PMC5396937

[zoo21894-bib-0094] Woc Colburn, A. M. , C. R. Sanchez , S. Citino , et al. 2018. “Cheetahs: Biology and Conservation.” In Cheetahs: Biology and Conservation. Biodiversity of the World, Conservation From Genes to Landscapes, 1st ed., edited by P. J. Nyhus , L. Marker , L. K. Boast , and A. Schmidt‐Kuentzel , 335–347. Academic Publisher. 10.1016/B978-0-12-804088-1.00024-1.

[zoo21894-bib-0095] Ziegler‐Meeks, K. , 2009. Husbandry Manual For The Cheetah.

