# Supplementary material for: What Makes a Mother? Investigating Maternal Success in Ex Situ Cheetahs
Source: Zoo Biol. 2025 Apr 13;44(4):332–44. doi: 10.1002/zoo.21894 (PMC12335232; doi:10.1002/zoo.21894)
Supplement: Supplementary file 1 — Supporting information. [file ZOO-44-332-s001.docx]

**Supplementary materials**

Indicating significant and non-significant variables tested in this study against litter size.

| **Variables** | **Univariable analysis** | | |
| --- | --- | --- | --- |
|  | Rates Ratio  (95% Confidence Interval) | *n* | *p-value* |
| **Maternal age at birth of litter** | **0.935 (0.900-0.971)** | ***36*** | **<0.001** |
| **Maternal line generations from wild** | 1.072 (0.940-1.221) | *32* | 0.300 |
| **Mother’s first litter** | 0.890 (0.739-1.072) | *35* | 0.221 |
| **Mother usually lives within eyesight and/or touch of male cheetahs*** | 0.840 (0.647- 1.090) | *35* | 0.190 |
| **Mother usually lives within eyesight and/or touch of female cheetahs*** | 1.174 (0.899-1.532) | *35* | 0.239 |
| **Mother usually lives within eyesight and/or touch of other cheetahs throughout the year*** | 1.097 (0.837-1.439) | *35* | 0.502 |
| **Mother remains in her usual enclosure during pregnancy and parenting** | 1.252 (0.947-1.655) | *38* | 0.114 |
| **Training practiced** | 0.892 (0.686- 1.159) | *38* | 0.392 |
| **Training practiced specifically for veterinary procedures (not crate, weight or recall)** | 0 .998 (0.770- 1.293) | *38* | 0.989 |
| **Artificial elevation platforms provided in enclosure** | **0.787 (0.619-0.999)** | ***38*** | **0.049** |
| **Mother cheetah receives ruminant meat every fortnight at minimum** | **1.349 (1.078-1.688)** | ***37*** | **0.009** |
| **Mother cheetah receives avian meat every fortnight at minimum** | 0.944 (0.714-1.250) | *37* | 0.689 |
| **Starve days practiced**** | 0.905 (0.696-1.177) | *37* | 0.456 |
| **Feederballs are not used as enrichment** | **1.474 (1.166-1.863)** | ***29*** | **0.001** |
| **Mechanical lures are not used as enrichment** | 1.176 (0.897-1.543) | *31* | 0.241 |
| **Fishing rods are not used as enrichment** | 1.150 (0.729-1.815) | *27* | 0.548 |
| **Novel scents are either never/rarely used as enrichment***** | **1.301 (1.014-1.670)** | ***35*** | **0.038** |
| **Novel objects are either never/rarely used as enrichment***** | **1.381 (1.091-1.747)** | ***32*** | **0.007** |
| **Catnip is occasionally used as an enrichment device** | 1.053 (0.763-1.452) | *28* | 0.754 |
| **Switching enclosure from usual enclosure to that of another cheetah and/or species is either never/rarely used as an enrichment device***** | 0.839 (0.647-1.087) | *36* | 0.184 |
| **Mother receives organised enrichment during pregnancy/parenting** | 0.784 (0.595-1.033) | *33* | 0.084 |
| **Mother receives organised enrichment during non-breeding times at most monthly** | 1.158 (0.873- 1.537) | *37* | 0.309 |
| **Mother receives organised enrichment during non-breeding times daily** | 0.875 (0.645- 1.185) | *37* | 0.388 |
| **Mate choice given****** | 1.115 (0.868-1.433) | *32* | 0.393 |
| **Discounting her own cubs living with her during the parenting period*  ***Starve days practiced during routine husbandry. Data insufficient to test starve days practiced during pregnancy and parenting specifically.*  **** Where “rarely” refers to something practiced less frequently than monthly*  *****Mate choice is provided through positive female response to the sight and/or smell of a male or where the female has actively broken out of her enclosure and sought the male herself* | | | |

Indicating significant and non-significant variables tested in this study against stillbirth occurrence.

| **Variables** | **Univariable analysis** | | |
| --- | --- | --- | --- |
|  | Odds Ratio  (95% Confidence Interval) | *n* | *p-value* |
| **Maternal age at birth of litter** | 1.166 (0.845- 1.607) | *36* | 0.350 |
| **Maternal line generations from wild** | 0.240 (0.055-1.053) | *32* | 0.059 |
| **Mother’s first litter** | 0.679 (0.323-1.426) | *35* | 0.307 |
| **Mother usually lives within eyesight and/or touch of male cheetahs*** | *Data insufficient to run model for this variable* | | |
| **Mother usually lives within eyesight and/or touch of female cheetahs*** | 0.217 (0.030- 1.573) | *35* | 0.131 |
| **Mother usually lives within eyesight and/or touch of other cheetahs throughout the year*** | *Data insufficient to run model for this variable* | | |
| **Mother remains in her usual enclosure during pregnancy and parenting** | **0.069 (0.013- 0.359)** | ***38*** | **0.001** |
| **Training practiced** | *Data insufficient to run model for this variable* | | |
| **Training practiced specifically for veterinary procedures** | 0.296 (0.039- 2.265) | *38* | 0.241 |
| **Artificial elevation platforms provided in enclosure** | *Data insufficient to run model for this variable* | | |
| **Mother cheetah receives ruminant meat every fortnight at minimum** | 0.217 (0.024-1.977) | *37* | 0.175 |
| **Mother cheetah receives avian meat every fortnight at minimum** | *Data insufficient to run model for this variable* | | |
| **Starve days practiced**** | **17.921 (1.861- 172.557)** | ***37*** | **0.013** |
| **Feederballs are not used as enrichment** | **0.094 (0.013-0.655)** | ***29*** | **0.017** |
| **Mechanical lures are not used as enrichment** | 1.784 (0.015- 217.283)  *Model fitness uncertain for this variable* | *31* | 0.813 |
| **Fishing rods are not used as enrichment** | *Data insufficient to run model for this variable* | | |
| **Novel scents are either never/rarely used as enrichment***** | **0.122 (0.019- 0.770)** | ***35*** | **0.025** |
| **Novel objects are either never/rarely used as enrichment***** | **0.122 (0.018-0.815)** | ***32*** | **0.030** |
| **Catnip is occasionally used as an enrichment device** | *Data insufficient to run model for this variable* | | |
| **Switching enclosure from usual enclosure to that of another cheetah and/or species is either never/rarely used as an enrichment device***** | 5.363 (0.605-47.537) | *36* | 0.131 |
| **Mother receives organised enrichment during pregnancy/parenting** | 3.674 (0.438-30.821) | *33* | 0.230 |
| **Mother receives organised enrichment during non-breeding times at most monthly** | 0.368 (0.047-2.862) | *37* | 0.339 |
| **Mother receives organised enrichment during non-breeding times daily** | *Data insufficient to run model for this variable* | | |
| **Mate choice given****** | *Data insufficient to run model for this variable* | | |
| **Discounting her own cubs living with her during the parenting period*  ***Starve days practiced during routine husbandry. Data insufficient to test starve days practiced during pregnancy and parenting specifically.*  **** Where “rarely” refers to something practiced less frequently than monthly*  *****Mate choice is provided through positive female response to the sight and/or smell of a male or where the female has actively broken out of her enclosure and sought the male herself* | | | |

Indicating significant and non-significant variables tested in this study against short-term mothering success.

| **Variables** | **Univariable analysis** | | |
| --- | --- | --- | --- |
|  | Odds Ratio  (95% Confidence Interval) | *n* | *p-value* |
| **Maternal age at birth of litter** | 0.732 (0.443- 1.210) | *31* | 0.224 |
| **Maternal line generations from wild** | 0.583 (0.211-1.611) | *27* | 0.298 |
| **Mother’s first litter** | 0.230 (0.026-2.038) | *31* | 0.187 |
| **Mother usually lives within eyesight and/or touch of male cheetahs*** | 0.252 (0.058-1.089) | *30* | 0.065 |
| **Mother usually lives within eyesight and/or touch of female cheetahs*** | 0.761 (0.148-3.913) | *30* | 0.743 |
| **Mother usually lives within eyesight and/or touch of other cheetahs throughout the year*** | 0.393 (0.071-2.176) | *30* | 0.285 |
| **Mother remains in her usual enclosure during pregnancy and parenting** | 1.446 (0.270-7.756) | *33* | 0.667 |
| **Training practiced** | 0.250 (0.035-1.771) | *33* | 0.165 |
| **Training practiced specifically for veterinary procedures** | 1.182 (0.223-6.250) | *33* | 0.844 |
| **Artificial elevation platforms provided in enclosure** | 0.808 (0.173-3.768) | *33* | 0.786 |
| **Mother cheetah receives ruminant meat every fortnight at minimum** | 1.658 (0.339-8.110) | *32* | 0.533 |
| **Mother cheetah receives avian meat every fortnight at minimum** | 1.678 (0.334-8.422) | *32* | 0.530 |
| **Starve days practiced**** | 0.230 (0.033-1.603) | *32* | 0.138 |
| **Feederballs are not used as enrichment** | **15.510 (2.522-95.401)** | ***25*** | **0.003** |
| **Mechanical lures are not used as enrichment** | 0.930 (0.208-4.163) | *27* | 0.925 |
| **Fishing rods are not used as enrichment** | 0.377 (0.024-5.836) | *23* | 0.486 |
| **Novel scents are either never/rarely used as enrichment***** | 1.160 (0.212-6.353) | *31* | 0.864 |
| **Novel objects are either never/rarely used as enrichment***** | 3.325 (0.420-26.345) | *28* | 0.255 |
| **Catnip is occasionally used as an enrichment device** | 1.376 (0.214-8.837) | *24* | 0.736 |
| **Switching enclosure from usual enclosure to that of another cheetah and/or species is either never/rarely used as an enrichment device***** | 0.537 (0.107-2.692) | *31* | 0.450 |
| **Mother receives organised enrichment during pregnancy/parenting** | 1.256 (0.215-7.321) | *28* | 0.800 |
| **Mother receives organised enrichment during non-breeding times at most monthly** | 0.631 (0.110-3.625) | *32* | 0.605 |
| **Mother receives organised enrichment during non-breeding times daily** | 1.216 (0.268-5.519) | *32* | 0.800 |
| **Mate choice given****** | 3.778 (0.312- 45.761) | *27* | 0.296 |
| **Discounting her own cubs living with her during the parenting period*  ***Starve days practiced during routine husbandry. Data insufficient to test starve days practiced during pregnancy and parenting specifically.*  **** Where “rarely” refers to something practiced less frequently than monthly*  *****Mate choice is provided through positive female response to the sight and/or smell of a male or where the female has actively broken out of her enclosure and sought the male herself* | | | |

Indicating significant and non-significant variables tested in this study against long-term mothering success.

| **Variables** | **Univariable analysis** | | |
| --- | --- | --- | --- |
|  | Odds Ratio  (95% Confidence Interval) | *n* | *p-value* |
| **Maternal age at birth of litter** | 0.804 (0.416-1.556) | *29* | 0.518 |
| **Maternal line generations from wild** | 0.739 (0.285-1.918) | *26* | 0.535 |
| **Mother’s first litter** | 0.999 (0.458-2.178) | *29* | 0.999 |
| **Mother usually lives within eyesight and/or touch of male cheetahs*** | 0.308 (0.039-2.403) | *28* | 0.261 |
| **Mother usually lives within eyesight and/or touch of female cheetahs*** | 0.586 (0.002-228.543)  *Model fitness uncertain for this variable* | *28* | 0.861 |
| **Mother usually lives within eyesight and/or touch of other cheetahs throughout the year*** | 0.036 (0.000-10.192) | *28* | 0.248 |
| **Mother remains in her usual enclosure during pregnancy and parenting** | 1.029 (0.151-7.037) | *33* | 0.977 |
| **Training practiced** | **0.086 (0.011-0.682)** | ***31*** | **0.020** |
| **Training practiced specifically for veterinary procedures** | 0.464 (0.073- 2.961) | *31* | 0.417 |
| **Artificial elevation platforms provided in enclosure** | 1.039 (0.178-6.068) | *31* | 0.966 |
| **Mother cheetah receives ruminant meat every fortnight at minimum** | 3.699 (0.589-23.234) | *30* | 0.163 |
| **Mother cheetah receives avian meat every fortnight at minimum** | 1.862 (0.271-12.794) | *30* | 0.527 |
| **Starve days practiced**** | 0.351 (0.052- 2.387) | *30* | 0.284 |
| **Feederballs are not used as enrichment** | **37.211 (4.047-342.150)** | ***24*** | **0.001** |
| **Mechanical lures are not used as enrichment** | 5.078 (0.545-47.317) | *25* | 0.154 |
| **Fishing rods are not used as enrichment** | 0.261 (0.017-4.086) | *22* | 0.339 |
| **Novel scents are either never/rarely used as enrichment***** | 4.090 (0.598-27.973) | *29* | 0.151 |
| **Novel objects are either never/rarely used as enrichment***** | **9.837 (1.112-86.997)** | ***27*** | **0.040** |
| **Catnip is occasionally used as an enrichment device** | 1.156 (0.118-11.289) | *23* | 0.901 |
| **Switching enclosure from usual enclosure to that of another cheetah and/or species is either never/rarely used as an enrichment device***** | 1.038 (0.143-7.504) | *29* | 0.971 |
| **Mother receives organised enrichment during pregnancy/parenting** | *Data insufficient to run model for this variable* | | |
| **Mother receives organised enrichment during non-breeding times at most monthly** | 1.705 (0.237-12.264) | *30* | 0.596 |
| **Mother receives organised enrichment during non-breeding times daily** | 0.228 (0.031- 1.681) | *30* | 0.147 |
| **Mate choice given****** | 1.274 (0.491- 3.301)  *Model fitness uncertain for this variable* | *26* | 0.619 |
| **Discounting her own cubs living with her during the parenting period*  ***Starve days practiced during routine husbandry. Data insufficient to test starve days practiced during pregnancy and parenting specifically.*  **** Where “rarely” refers to something practiced less frequently than monthly*  *****Mate choice is provided through positive female response to the sight and/or smell of a male or where the female has actively broken out of her enclosure and sought the male herself* | | | |

Indicating significant and non-significant variables tested in this study against keeper-reported maternal negligence.

| **Variables** | **Univariable analysis** | | |
| --- | --- | --- | --- |
|  | Odds Ratio  (95% Confidence Interval) | *n* | *p-value* |
| **Maternal age at birth of litter** | 0.843 (0.544-1.307) | *35* | 0.446 |
| **Maternal line generations from wild** | 0.876 (0.486-1.581) | *31* | 0.661 |
| **Mother’s first litter** | 2.259 (0.607-8.417) | *34* | 0.224 |
| **Mother usually lives within eyesight and/or touch of male cheetahs*** | 0.638 (0.120-3.396) | *36* | 0.599 |
| **Mother usually lives within eyesight and/or touch of female cheetahs*** | 0.277 (0.050-1.518) | *36* | 0.139 |
| **Mother usually lives within eyesight and/or touch of other cheetahs throughout the year*** | 0.638 (0.146-2.791) | *36* | 0.550 |
| **Mother remains in her usual enclosure during pregnancy and parenting** | **0.120 (0.022-0.644)** | ***37*** | **0.013** |
| **Training practiced** | 0.495 (0.112-2.193) | *37* | 0.354 |
| **Training practiced specifically for veterinary procedures** | 0.419 (0.078-2.250) | *37* | 0.310 |
| **Artificial elevation platforms provided in enclosure** | 0.667 (0.148-3.002) | *37* | 0.598 |
| **Mother cheetah receives ruminant meat every fortnight at minimum** | 0.887 (0.202-3.891) | *36* | 0.873 |
| **Mother cheetah receives avian meat every fortnight at minimum** | 1.183 (0.255-5.490) | *36* | 0.830 |
| **Starve days practiced**** | 1.768 (0.370-8.457) | *36* | 0.475 |
| **Feederballs are not used as enrichment** | 0.523 (0.116-2.370) | *28* | 0.401 |
| **Mechanical lures are not used as enrichment** | 1.434 (0.370-5.566) | *30* | 0.602 |
| **Fishing rods are not used as enrichment** | 0.350 (0.084-1.450) | *26* | 0.148 |
| **Novel scents are either never/rarely used as enrichment***** | 0.604 (0.113- 3.231) | *34* | 0.556 |
| **Novel objects are either never/rarely used as enrichment***** | 0.572 (0.110-2.981) | *31* | 0.507 |
| **Catnip is occasionally used as an enrichment device** | **3.036 (1.306-7.059)** | ***27*** | **0.010** |
| **Switching enclosure from usual enclosure to that of another cheetah and/or species is either never/rarely used as an enrichment device***** | 1.369 (0.331-5.662) | *35* | 0.665 |
| **Mother receives organised enrichment during pregnancy/parenting** | 0.333 (0.071-1.564) | *34* | 0.164 |
| **Mother receives organised enrichment during non-breeding times at most monthly** | 2.285 (0.426-12.245) | *36* | 0.335 |
| **Mother receives organised enrichment during non-breeding times daily** | 1.012 (0.216-4.743) | *36* | 0.988 |
| **Mate choice given****** | 1.493 (0.246-9.048) | *33* | 0.663 |
| **Discounting her own cubs living with her during the parenting period*  ***Starve days practiced during routine husbandry. Data insufficient to test starve days practiced during pregnancy and parenting specifically.*  **** Where “rarely” refers to something practiced less frequently than monthly*  *****Mate choice is provided through positive female response to the sight and/or smell of a male or where the female has actively broken out of her enclosure and sought the male herself* | | | |

To access a digital draft of the survey sent to zoological collections for this research, please visit the link : <https://www.surveymonkey.com/r/Preview/?sm=ppXSxZjs1uOq_2BANWPxtT8ziwyCXyONeMkjmW4y8agXNU94DPGtkktwv5bWYqzXej&state=invite_modal>
